# Effect of renal dysfunction in dogs on the disposition and marrow toxicity of melphalan.

**DOI:** 10.1038/bjc.1981.52

**Published:** 1981-03

**Authors:** D. S. Alberts, H. G. Chen, D. Benz, N. L. Mason

## Abstract

The effect of renal failure on melphalan pharmacology and toxicity has been poorly understood. Such information is of interest because melphalan is the most commonly used anticancer drug in the treatment of multiple myeloma, which is frequently associated with renal failure. We have studied the disposition and marrow toxicity of parenteral melphalan in dogs before and after induction of renal failure with subtotal nephrectomy. The surgical procedure decreased the creatinine clearance by an average of 62% (P = 0.001). The lowest neutrophil counts following i.v. melphalan (1 mg/kg) averaged 4.9 x 10(3)/mm3 pre-nephrectomy and 0.9 x 10(3)/mm3 post-nephrectomy, respectively (P = 0.002). The mean lowest recorded platelet counts after melphalan (1 mg/kg) were 115 x 10(3)/mm3 in the pre-nephrectomized dogs, and 9.7 x 10(3/mm3 in those who had been nephrectomized (P = 0.002). Following nephrectomy, i.v. melphalan's terminal-phase plasma half-life and renal clearance were both raised (P = 0.02) to 75% over pre-nephrectomy values. These studies show that i.v. melphalan-induced myelosuppression is markedly increased and its plasma elimination and renal clearance significantly decreased in the presence of renal dysfunction in dogs. These data suggest that parenteral melphalan's starting dose be decreased by at least 50% when used in myeloma patients with renal failure.


Br. J. Cancer (1981) 43, 330

### EFFECT OF RENAL DYSFUNCTION IN DOGS ON THE DISPOSITION AND MARROW TOXICITY OF MELPHALAN

D. S. ALBERTS, H.-S. G. CHEN*, D. BENZ AND N. L. MASON

*From the Section of Hematology/Oncology, Department of Internal Medicine, and the Cancer Center, College of Medicine, University of Arizona, Tucson, AZ 85724 and*
**Department of Chemical Engineering, University of Arizona, Tucson, AZ 85721, U.S.A.*

Received 30 September 1980 Accepted 17 November 1980

**Summary.—The effect of renal failure on melphalan pharmacology and toxicity has been poorly understood. Such information is of interest because melphalan is the most commonly used anticancer drug in the treatment of multiple myeloma, which is frequently associated with renal failure. We have studied the disposition and marrow toxicity of parenteral melphalan in dogs before and after induction of renal failure with subtotal nephrectomy. The surgical procedure decreased the creatinine clearance by an average of 62% ($P=0{\cdot}001$). The lowest neutrophil counts following i.v. melphalan (1 mg/kg) averaged $4{\cdot}9\times10^3/mm^3$ pre-nephrectomy and $0{\cdot}9\times10^3/mm^3$ post-nephrectomy, respectively ($P=0{\cdot}002$). The mean lowest recorded platelet counts after melphalan (1 mg/kg) were $115\times10^3/mm^3$ in the pre-nephrectomized dogs, and $9{\cdot}7\times10^3/mm^3$ in those who had been nephrectomized ($P=0{\cdot}002$). Following nephrectomy, i.v. melphalan's terminal-phase plasma half-life and renal clearance were both raised ($P=0{\cdot}02$) to 75% over pre-nephrectomy values. These studies show that i.v. melphalan-induced myelosuppression is markedly increased and its plasma elimination and renal clearance significantly decreased in the presence of renal dysfunction in dogs. These data suggest that parenteral melphalan's starting dose be decreased by at least 50% when used in myeloma patients with renal failure.**

MELPHALAN continues to be one of the most important anticancer drugs in the treatment of multiple myeloma (Speed *et al.*, 1964; Alexanian *et al.*, 1968; Bergsagel *et al.*, 1979). As many as one third of all myeloma patients will have severely reduced renal function as a result of renal tubular damage from paraprotein deposition and/or the toxic effects of hypercalcaemia (Kayle & Bayrd, 1976). Although the alkylating agent melphalan is commonly used in myeloma patients with renal failure, it is not known whether renal dysfunction alters its pharmacokinetics or marrow toxicity. Speed *et al.* (1964) suggested that the rate of melphalan urinary excretion might be decreased in the presence of renal failure. We have studied the disposition and marrow toxicity of melphalan in dogs before and after surgically induced renal failure. The results of these studies suggest that severe renal dysfunction can slow the plasma and renal clearance of melphalan and markedly increase neutropenia and thrombocytopenia.

### MATERIALS AND METHODS

*Dogs.*—Eight male mongrel dogs, weighing 17–23 kg, which had been previously quarantined for 2–4 weeks in the vivarium, were used. The dogs were without evidence of infection and were not given other drugs during the entire duration of the melphalan studies. Baseline BUN, serum creatinine, and 24h urinary creatinine clearance were measured in each of the animals, and were remeasured after the surgical induction of renal dysfunction.

An indwelling 16-gauge catheter was inserted into a superficial leg vein. Melphalan (1 mg/kg body weight) was injected i.v. over about 1 min, followed by 10 ml normal saline. The catheter was thoroughly flushed several times with blood after melphalan injection and blood samples were taken through this catheter.

*Surgical procedures*.—Four to six weeks after the first dose of melphalan, azotemia was produced by unilateral total nephrectomy and contralateral partial nephrectomy. Gierke *et al*., 1978. The right kidney was removed through a midabdominal incision. Branches of the contralateral renal artery were ligated and the extent of necrosis was determined by observing ischemia on the surface of the kidney until about one quarter of the remaining kidney remained viable. If the arterial branching at the renal pelvis was not sufficiently extensive to accomplish this, part of the kidney was ligated with number 0 chromic gut after the capsule was lifted from the ligated segment of renal parenchyma. The capsule was sewn over the ligated portion of the kidney parenchyma to control bleeding. The experimental protocol previously described (*i.e*., melphalan, 1 mg/kg i.v.) was repeated when serum electrolytes, creatinine and blood urea nitrogen (BUN) had stabilized.

*Drug formulation, dosage and administration*.—Melphalan powder (Alkeran, Burroughs Wellcome Co., Research Triangle Park, NC) was stored in powder form at −4°C until use. Immediately before administration, 1 ml of Burroughs Wellcome acid ethanol was added to 100 mg melphalan, which after dissolution was further diluted with 9 ml of Burroughs Wellcome brand diluent (*i.e*., $K_2HPO_4$ in propylene glycol and water) and placed on ice.

*Blood and urine sampling*.—Blood samples (10 ml) were collected in tubes containing 100 iu of heparin. Blood samples were taken just before the start of therapy and at 5, 15, 30, 45 and 60 min, and 2, 3, 4, 6, 8 and 24 h after drug injection. They were placed on ice for a short period before refrigerated centrifugation at 2000 rev/min for 10 min. The separated plasma samples were then stored at −20°C. Fractional urine collection, using size-8 straight, disposable catheters, were taken for the first 6 h after drug injection. The dogs were then placed in metabolic cages and urine was collected for 24h periods up to 3 days. The urine was drained from the bottom of the cage into collection bottles. The urinary volume of each 24h period was measured and aliquots were stored at −20°C in sterile containers to which concentrated HCl had been added as a preservative.

*Blood counts and serum chemistries*.—Standard procedures were used to measure renal function and complete blood counts on the dogs at weekly intervals to evaluate renal status and melphalan's haemopoietic toxicity.

*Melphalan assays*.—Melphalan was assayed in biological fluids using high-pressure liquid chromatography (HPLC) as previously described by Chang *et al*. (1978).

*Data analysis*.—The concentrations of melphalan in plasma were averaged to obtain the combined data for the pre- and post-nephrectomy groups, respectively. Each dog's data and the combined data for each group were then fitted to a multiexponential equation using a nonlinear-regression computer program, NONLIN (Metzler, 1969). The half-lives and area under the plasma decay curve (C×T) were calculated from the parameters generated by curve fitting (Alberts *et al*., 1979*a*). The melphalan renal clearances (Qr) were calculated from:

$$Qr = \frac{\text{total urinary excretion}}{C \times T}$$

for each dog and then averaged for both pre- and post-nephrectomy groups.

To compare the effect of nephrectomy on melphalan pharmacokinetics, a two-tailed, unpaired Student's *t* test was used to compute the *P* values for the important parameters. A *P* of $<0{\cdot}05$ was taken as statistically significant.

### RESULTS

### *Renal status*

The BUN, serum creatinine and 24h urinary creatinine clearance before nephrectomy for 8 dogs averaged $19{\cdot}9 \pm 5{\cdot}3$ mg/100 ml, $1{\cdot}0 \pm 0{\cdot}02$ mg/100 ml, and $41{\cdot}2 \pm 2{\cdot}7$ ml/min, respectively (Table I). After surgical induction of renal failure, BUN increased more than 2-fold, serum creatinine more than 3-fold, and 24h urinary creatinine clearance decreased to less than half (Table I). For each laboratory test pre- and post-nephrectomy values were statistically different, as shown in Table I.

### *Melphalan-induced myelosuppression*

In the dogs with normal renal function, melphalan (1 mg/kg i.v.) produced the

TABLE I.—*Measurements of renal function in dogs before and after surgically induced renal failure (mean ± s.e.)*

| | BUN (mg%) | Creatinine (mg%) | Creatinine clearance (ml/min) |
|---|---|---|---|
| Pre-nephrectomy | 19·9 ± 5·3 | 1·0 ± 0·02 | 41·2 ± 2·7 |
| Post-nephrectomy | 49·8 ± 5·3 | 3·2 ± 0·41 | 15·7 ± 7·6 |
| *P* | 0·004 | 0·001 | 0·001 |

following average nadir blood counts on Day 14, as shown in Table II: WBC 5·1 ± 1·5 × 10³/mm³; neutrophils 4·9 ± 0·7 × 10³/mm³; and platelet count 115 ± 23 × 10³/mm³. There was no evidence that repeated doses of i.v. melphalan (*i.e.* 1 mg/kg) at monthly intervals for up to 3 consecutive courses induced cumulative marrow damage in either normal or nephrectomized dogs. After the surgical induction of renal dysfunction, i.v. melphalan caused a more than 2-fold reduction in the average nadir white cell count, a more than 5-fold reduction in the mean nadir neutrophil count and a more than 11-fold reduction in the mean nadir platelet count. For neutrophils and platelets, pre- and post-nephrectomy values were statistically different (Table II). After nephrectomy, the neutrophil and platelet count nadirs were on Day 8 (*i.e.* 6 days before the pre-nephrectomy drug-induced nadirs).

TABLE II.—*Melphalan myelotoxicity following 1 mg/kg i.v. in dogs before and after surgically induced renal failure (mean ± s.e.)*

| | WBC (×10³/mm³) | Neutrophils (×10³/mm³) | Platelets (×10³/mm³) |
|---|---|---|---|
| Pre-nephrectomy | 5·1 ± 1·5 | 4·9 ± 0·7 | 115·0 ± 23·0 |
| Post-nephrectomy | 2·0 ± 0·4 | 0·9 ± 0·2 | 9·7 ± 3·0 |
| *P* | 0·08 | 0·001 | 0·002 |

PHARMACOKINETIC PARAMETERS

*Melphalan plasma kinetics*

Prior to the surgical induction of renal dysfunction, the normal dogs had a melphalan $t_{\frac{1}{2}}^{\beta}$ of 43·5 ± 3·1 min and an average C × T of 51·8 μg.min/ml (Table III). After renal dysfunction the melphalan

FIG. 1.—Melphalan (HPLC) plasma-disappearance curves before and after the induction of renal failure in dogs. Data points represent the average of the melphalan plasma concentration at each time point for up to 8 dogs. Lower curve (○) dogs prior to nephrectomy. Upper curve (●) nephrectomized dogs.

TABLE III.—*Pharmacokinetics of i.v. melphalan (HPLC) before and after surgically induced renal failure in dogs*

| | Plasma | | | Urine | |
|---|---|---|---|---|---|
| | $t_{\frac{1}{2}}^{\alpha}$ (min) | $t_{\frac{1}{2}}^{\beta}$ (min) | C × T (μg.min/ml) | 24h urinary excretion (%) | Renal clearance (ml/min) |
| Pre-nephrectomy | 4·5 ± 2·0 | 43·5 ± 3·1 | 51·8 | 4·6 ± 0·9 | 23·6 ± 3·0 |
| Post-nephrectomy | 4·7 ± 2·5 | 76·2 ± 21·9 | 81·0 | 3·1 ± 0·5 | 13·2 ± 2·2 |
| *P* | Not sig. | 0·017 | Not sig. | 0·14 | 0·02 |

* C × T = area under the plasma disappearance curve.

plasma $t_{\frac{1}{2}}{}^{\beta}$ was $76 \cdot 2 \pm 12 \cdot 9$ min and average $C \times T$, 81 $\mu$g.min/ml. Melphalan's plasma $t_{\frac{1}{2}}{}^{\beta}$ was significantly prolonged ($P = 0 \cdot 017$) when computed from only terminal-slope data.

Fig. 1 shows the plasma disappearance curves for melphalan (1 mg/kg i.v. bolus) before and after surgical induction of renal dysfunction. Note that the upper curve is that associated with melphalan given to dogs with renal dysfunction.

*Urinary excretion and clearance data*

Fig. 2 shows the curves describing the rate of urinary excretion of melphalan (1 mg/kg i.v. bolus) in dogs before and after the surgical induction of renal dysfunction. Markedly lower melphalan concentrations were seen in the dogs with renal impairment at 10–64 h after melphalan administration. Although melphalan's 24h urinary-excretion percentage was not significantly changed, its renal clearance was statistically significant between pre- and post-nephrectomy ($P = 0 \cdot 02$) (Table III).

FIG. 2.—Melphalan (HPLC) urinary excretion rates before and after the induction of renal failure in dogs. Data points represent the average melphalan urinary concentrations for up to 8 dogs. Lower curve (●) represents nephrectomized dogs. Upper curve (○) represents dogs prior to nephrectomy.

### DISCUSSION

We have used a well-standardized dog model (Gierke *et al.*, 1978) to study the effect of renal dysfunction on the toxicity and disposition of melphalan, because it had not been directly investigated either in the clinic or in a model system. Furner *et al.* (1977) have previously described the pharmacokinetics of i.v. melphalan in the dog. Their melphalan plasma disappearance and urinary excretion data are similar to those we observed in our study dogs before nephrectomy. After the surgical induction of renal dysfunction there was a 75% rise in melphalan's terminal-phase plasma disappearance and renal clearance. These statistically significant changes in drug disposition were associated with a 5-fold reduction in the mean nadir neutrophil count and an 11-fold reduction in the mean nadir platelet count. These data suggest that the initial dose of parenteral melphalan should be markedly reduced in patients with severe renal failure.

Unfortunately, our dog model cannot be used to determine exact dose adjustments for i.v. melphalan in patients with reduced renal function. In the face of a drug-induced severe or life-threatening neutropenia (*i.e.* $<500$ to $1000/mm^3$ respectively) or thrombocytopenia (*i.e.* $<25{,}000$–$50{,}000/mm^3$) the subsequent dose of the responsible drug is usually reduced by at least 50%. A reasonable clinical guideline for i.v. melphalan dosage adjustment in patients with severe renal disease would be an initial 50% reduction of the standard dose, with careful dose escalation in subsequent courses, as tolerated. Such guidelines have been used by the Cancer and Leukemia Group B in a randomized study of i.v. bolus melphalan in myeloma patients who have varying degrees of renal failure (Corwell, personal communication).

We do not recommend a dose reduction of orally administered melphalan in myeloma patients with diminished renal function. We have previously shown that there is marked variability of systemic availability of melphalan after oral administration (Alberts *et al.*, 1979*b*), and the oral route would tend to minimize the adverse marrow effects. When the parenteral route is used, however, the suggested dose adjustments should be made.

Supported by research grants CA-17094 and T32-GM07533 from the National Institutes of Health, U.S. Public Health Service, Department of Health, Education, and Welfare, Bethesda, MD; Burroughs Wellcome Co., Research Triangle Park, NC; and the Phi Beta Psi National Sorority, Lima, OH.

We wish to thank Dr Klaus Gierke for his surgical expertise, Dr Sai Y. Chang and Barbara Larcom for their analytical chemistry expertise, Tom Evans and Robin Staerkel for their research assistance, and Dr Sydney E. Salmon for his scientific advice and critical review of this manuscript.

### REFERENCES

Alberts, D. S., Chang, S. Y., Chen, H.-S.G. & others (1979*a*) Kinetics of intravenous melphalan. *Clin. Pharmacol. Ther.*, **26**, 73.

Alberts, D. S., Chang, S. Y., Chen, H.-S.G., Evans, T. L. & Moon, T. E. (1979*b*) Oral melphalan kinetics. *Clin. Pharmacol. Ther.*, **26**, 737.

Alexanian, R., Bergsagel, D. E., Migliore, P. J., Vaughn, W. K. & Howe, C. D. (1968) Melphalan therapy for plasma cell myeloma. *Blood*, **31**, 1.

Bergsagel, D., Bailey, A. J., Langley, G. R., McDonald, R. N., White, D. F. & Miller, A. B. (1979) The chemotherapy of plasma-cell myeloma and the incidence of acute leukemia. *N. Engl. J. Med.*, **301**, 743.

Chang, S. Y., Alberts, D. S., Melnick, L. R. & others (1978) High-pressure liquid chromatographic analysis of melphalan in plasma. *J. Pharm. Sci.*, **67**, 679.

Furner, R. L., Borwn, R. K. & Duncan, G. (1977) Pharmacokinetics of absorption, distribution and elimination of melphalan in the dog. *Cancer Treat. Rep.*, **61**, 1637.

Gierke, K. D., Perrier, D., Mayersohn, M. & Marcus, F. I. (1978) Digoxin disposition kinetics in dogs before and during azotemia. *J. Pharm. Exp. Therap.*, **205**, 459.

Kayle, R. A. & Bayrd, E. D. (1976) *The Monoclonal Gammopathies: Multiple Myeloma and Related Disorders*. Springfield: Charles C. Thomas. p. 91.

Metzler, C. M. (1969) NONLIN: A computer program for parameter estimation in nonlinear situations. Kalamazoo: Upjohn Co. *Technical Report* 7292/69/7292/005.

Speed, D. E., Galton, D. A. G. & Swan, A. (1964) Melphalan in the treatment of myelomatosis. *Br. Med. J.*, **1**, 1664.


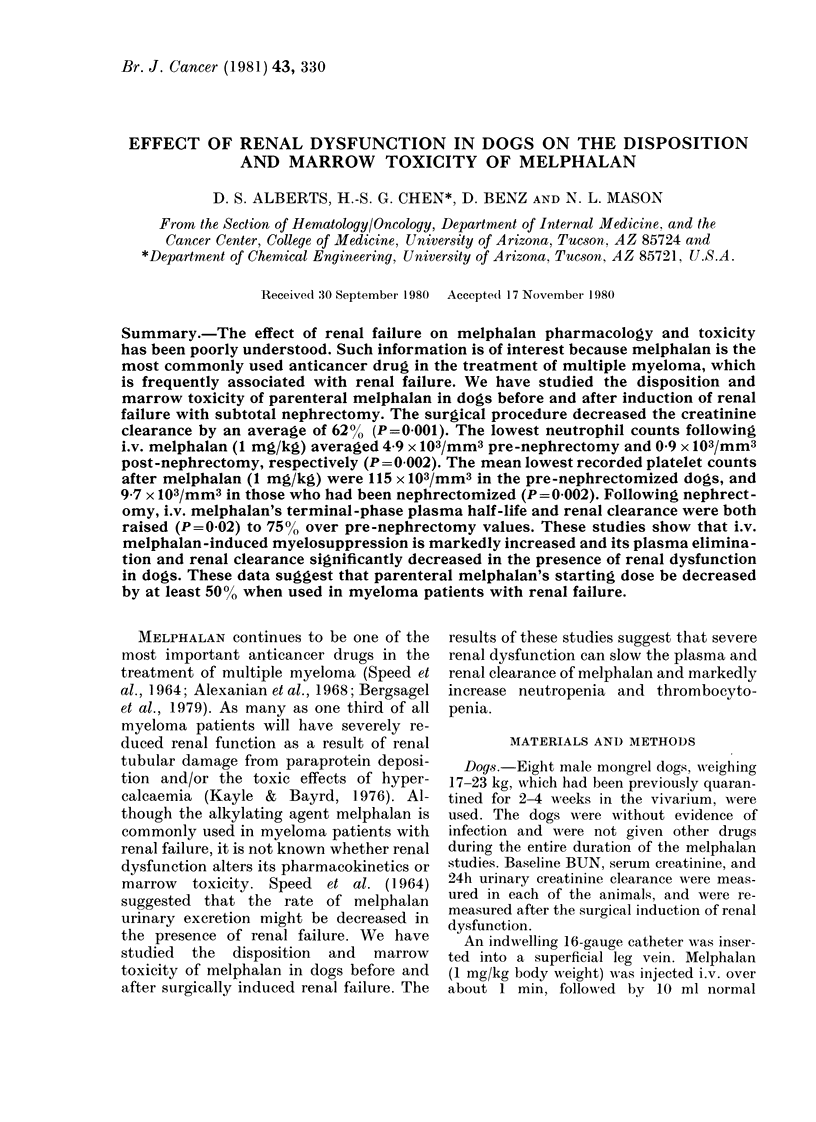

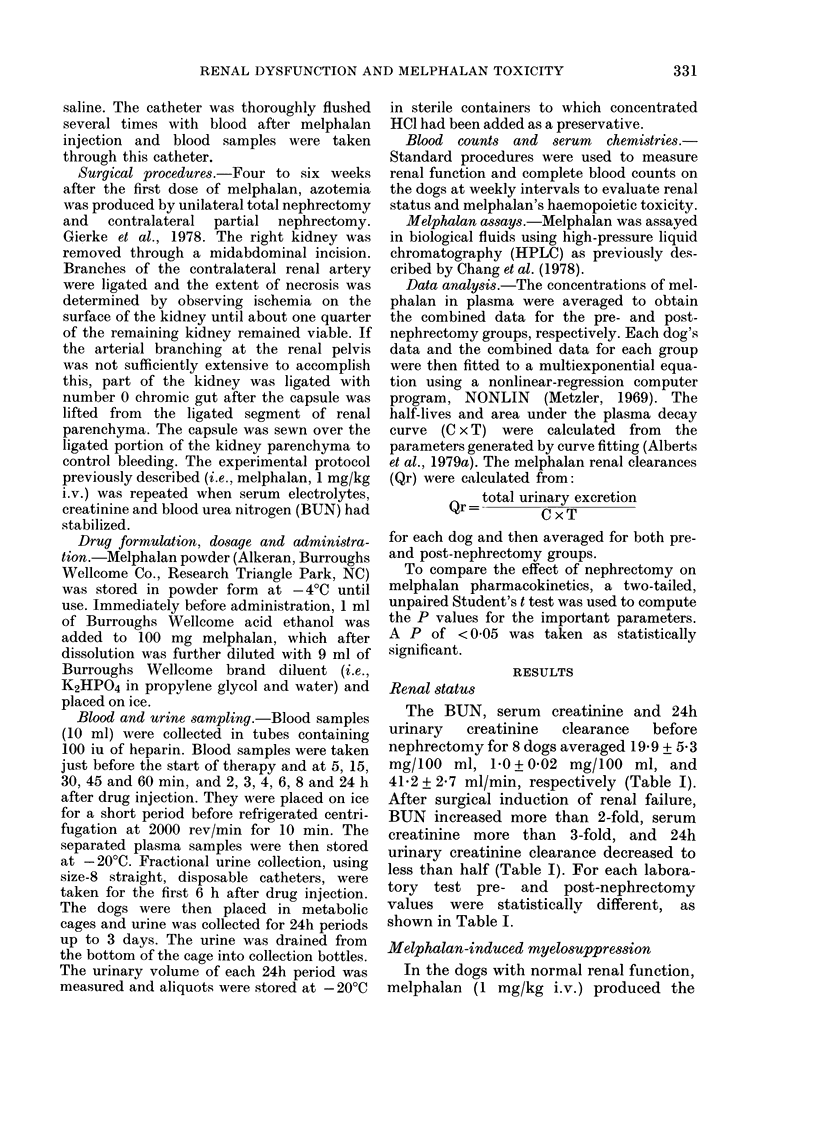

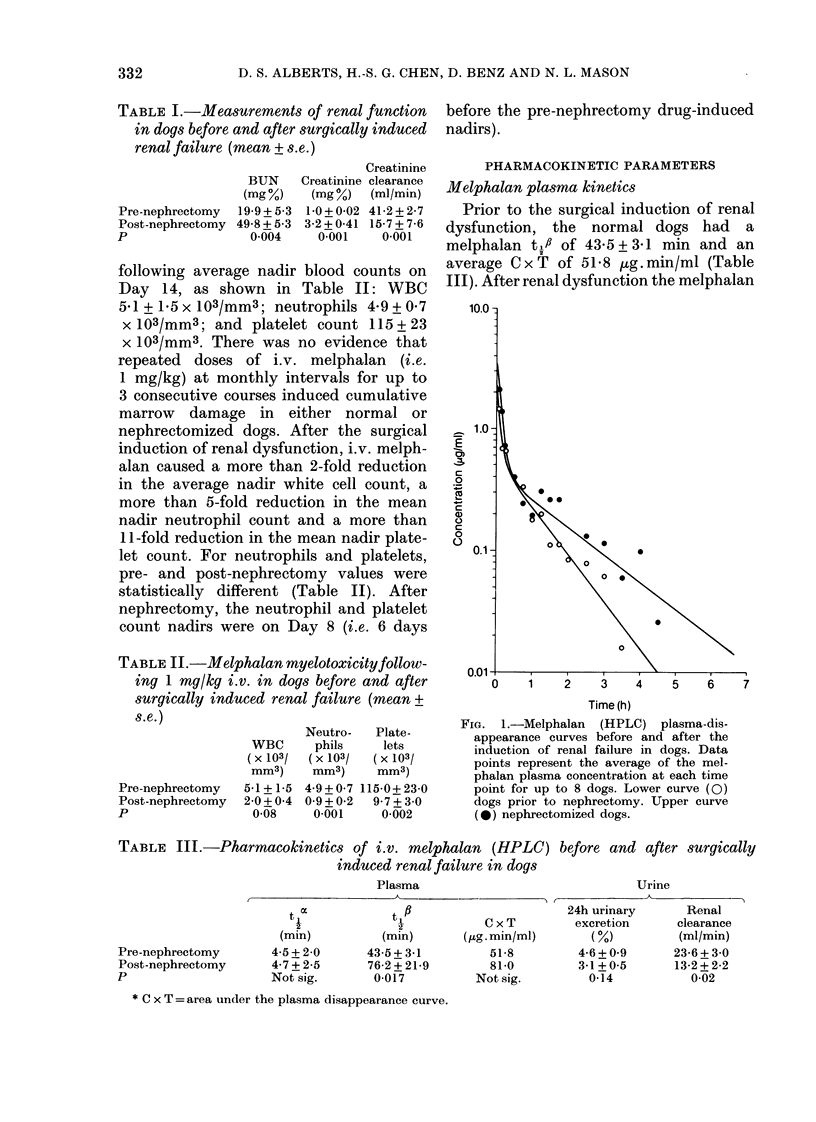

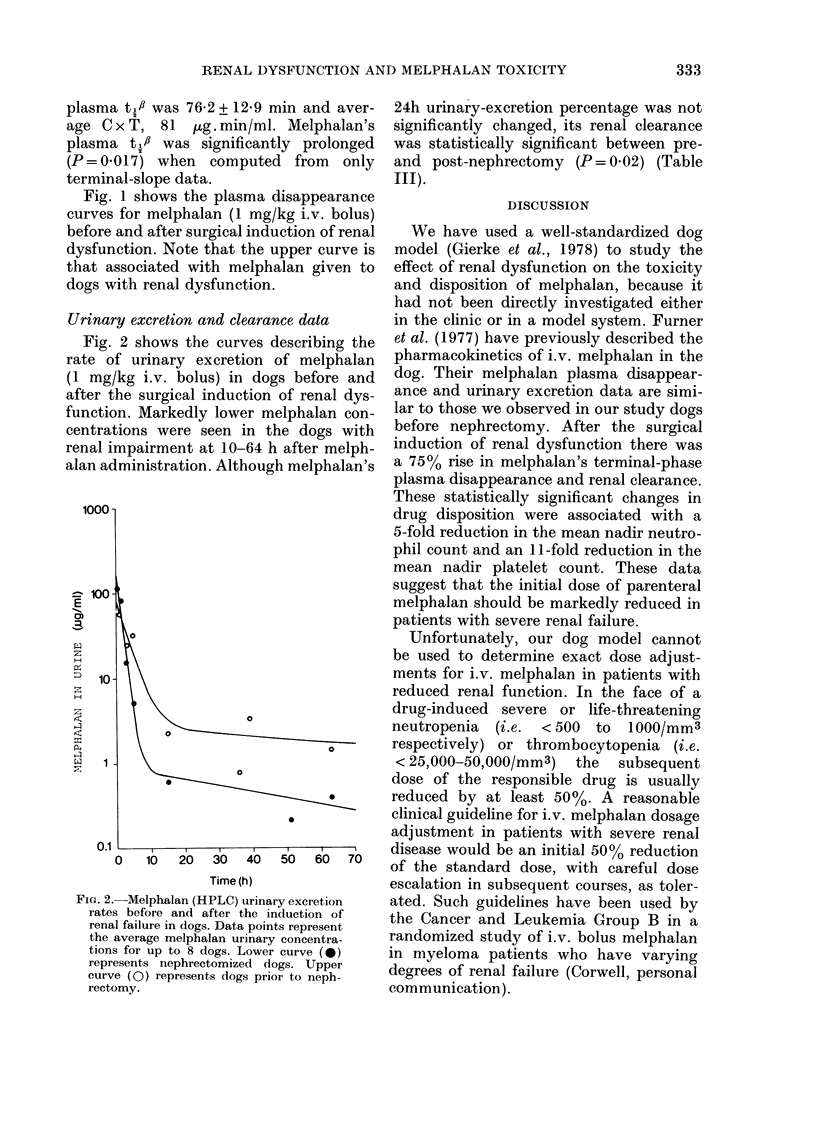

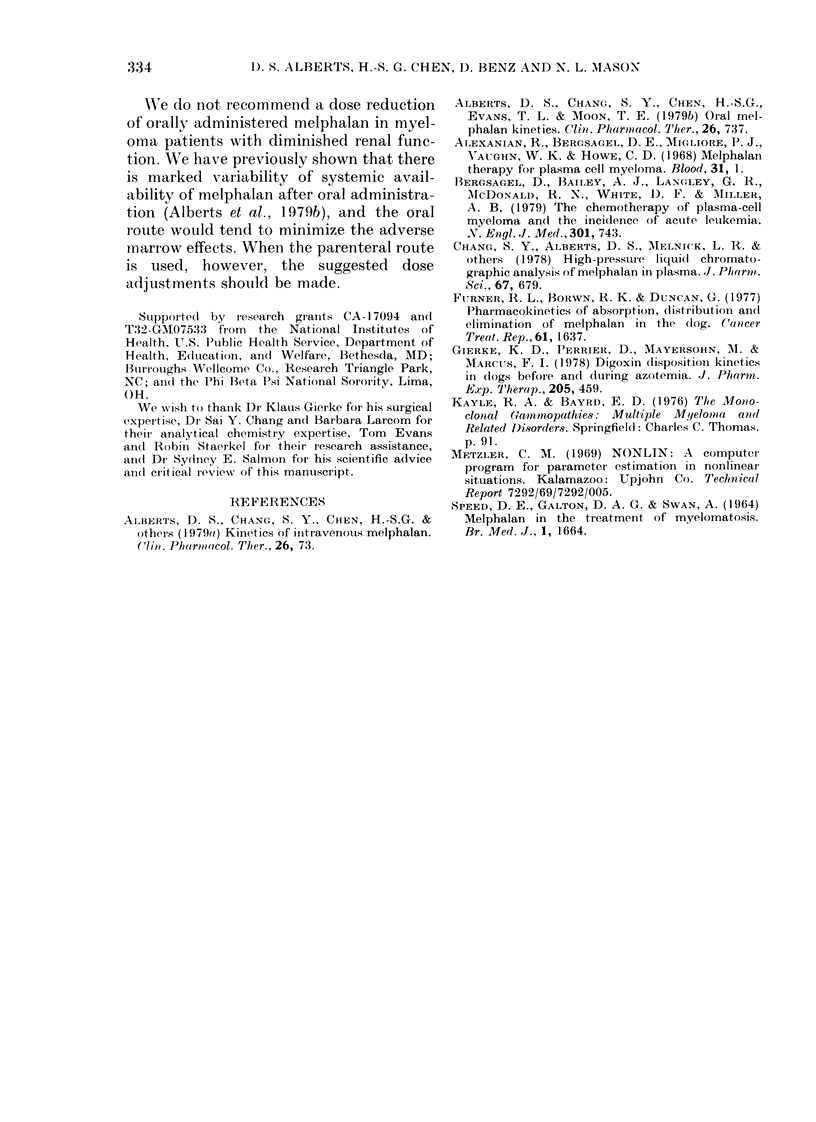


## References

[OCR_00495] Alberts D. S., Chang S. Y., Chen H. S., Evans T. L., Moon T. E. (1979). Oral melphalan kinetics.. Clin Pharmacol Ther.

[OCR_00500] Alexanian R., Bergsagel D. E., Migliore P. J., Vaughn W. K., Howe C. D. (1968). Melphalan therapy for plasma cell myeloma.. Blood.

[OCR_00512] Chang S. Y., Alberts D. S., Melnick L. R., Walson P. D., Salmon S. E. (1978). High-pressure liquid chromatographic analysis of melphalan in plasma.. J Pharm Sci.

[OCR_00518] Furner R. L., Brown R. K., Duncan G. (1977). Pharmacokinetics of the absorption, distribution, and elimination of melphalan in the dog.. Cancer Treat Rep.

[OCR_00526] Gierke K. D., Perrier D., Mayersohn M., Marcus F. I. (1978). Digoxin disposition kinetics in dogs before and during azotemia.. J Pharmacol Exp Ther.

[OCR_00542] SPEED D. E., GALTON D. A., SWAN A. (1964). MELPHALAN IN THE TREATMENT OF MYELOMATOSIS.. Br Med J.

